# Characteristics of Cannabis-Only and Other Drug Users Who Visit the Emergency Department

**DOI:** 10.1089/can.2016.0012

**Published:** 2016-07-01

**Authors:** Susan I. Woodruff, Cameron T. McCabe, Melinda Hohman, John D. Clapp, Audrey M. Shillington, Kimberly Eisenberg, C. Beth Sise, Edward M. Castillo, Theodore C. Chan, Michael J. Sise

**Affiliations:** ^1^ Center for Alcohol and Drug Studies and Services, School of Social Work, San Diego State University, San Diego, California.; ^2^ Department of Psychology, Portland State University, Portland, Oregon.; ^3^ School of Social Work, San Diego State University, San Diego, California.; ^4^ College of Social Work, The Ohio State University, Columbus, Ohio.; ^5^ School of Social Work, Colorado State University, Fort Collins, Colorado.; ^6^ San Diego State University Research Foundation, San Diego, California.; ^7^ Trauma Services [MER-62], Scripps Mercy Hospital, San Diego, California.; ^8^ Department of Emergency Medicine, University of California San Diego, San Diego, California.

**Keywords:** addiction, behavior, drug abuse

## Abstract

Emergency department (ED) settings have gained interest as venues for illegal drug misuse prevention and intervention, with researchers and practitioners attempting to capitalize on the intersection of need and opportunity within these settings. This study of 686 adult patients visiting two EDs for various reasons who admitted drug use compared daily cannabis-only users, nondaily cannabis-only users, and other drug users on sociodemographic and drug-related severity outcomes. The three drug use groups did not differ on most sociodemographic factors or medical problem severity scores. Forty-five percent of the sample was identified as having a drug use problem. ED patients who used drugs other than cannabis were at particular risk for high drug use severity indicators and concomitant problems such as psychiatric problems and alcohol use severity. However, 19–29% of cannabis-only users were identified as having problematic drug use. Furthermore, daily cannabis-only users fared less well than nondaily cannabis users with regard to drug use severity indicators and self-efficacy for avoiding drug use. Results may assist emergency medicine providers and medical social workers in matching patients to appropriate intervention. For example, users of drugs other than cannabis (and perhaps heavy, daily cannabis-only users) may need referral to specialty services for further assessment. Enhancement of motivation and self-efficacy beliefs could be an important target of prevention and treatment for cannabis-only users screened in the ED.

## Introduction

In the United States, the annual cost of drug abuse to society related to lost productivity, crime, and healthcare is estimated to be $193 billion.^1^ Such use places a significant burden on the healthcare system partly because drug users (including cannabis users) are more likely than nondrug users to use the emergency department (ED) as a primary source of medical care.^2–5^ ED settings have gained interest as venues for drug abuse prevention and intervention, with researchers and practitioners attempting to capitalize on the intersection of need and opportunity within these settings.^5^

Cannabis use in the United States has steadily increased over the past decade, whereas use of many other substances has declined.^6^ Those who use cannabis exclusively are typically in better health overall than users of other drugs, although cannabis use can be associated with both acute and chronic psychosocial, mental, and physical health consequences, including cancer and long-term respiratory and cardiovascular problems.^7–10^

With the passage of less punitive measures or outright legalization of cannabis use for medical and/or recreational use in 23 states, as well as mounting evidence of dose-dependent psychocognitive health effects of cannabis,^11,12^ more information is needed about users of cannabis only and how they compare with other drug users who present to the ED. Such efforts may shed light on differences in drug use patterns and associated health effects, and provide important information for future intervention efforts. This study examined the sociodemographic characteristics, drug use severity, and problems related to drug use among ED patients who were daily versus nondaily cannabis-only users, as well as those who reported noncannabis drug use.

## Materials and Methods

### Procedures

Participants were 686 consecutively enrolled adult patients visiting the EDs and trauma units of two large urban safety net hospitals in Southern California who reported to trained paraprofessional health interviewers that they had used illicit drugs, or legal drugs in a nonprescribed manner, during the past 30 days (additional details have been published elsewhere).^13,14^ Over a 1-year period, interviewers working during peak hours attempted to approach all capable adult patients, regardless of the reason for the patient's visit. Patients under the age of 18, those with severely altered mental status, those physically incapable of participating due to severe illness or injury, and those unable to speak English or Spanish were excluded from participation. After providing consent, as approved by our university and hospital IRBs, these patients were interviewed further to collect sociodemographic information, additional drug use data, and measures of problem areas related to drug use. Refusals to be screened by trained interviewers were rare.^14^

### Measures

Sociodemographic variables included participants' age; gender; race/ethnicity (Hispanic/Latino, white non-Latino, African American, and other); marital status (i.e., single, which included never married, divorced, separated, or widowed, vs. married/living as married); years of education; annual household income (measured by six categories ranging from less than $9999 to $50,000 or more); and whether or not the participant had been employed in the past 30 days.

Participants completed the ASI-Lite, a condensed version of the Addiction Severity Index.^15,16^ The ASI-Lite gathers quantitative information (i.e., number of days in the past 30 days) about the participant's recent use of several types of drugs (i.e., heroin, illicit methadone, other opiates/analgesics, barbiturates, sedatives/hypnotics/tranquilizers, cocaine, amphetamines, cannabis/marijuana, hallucinogens, and “other drugs”). Fifty-seven percent of participants (*n*=394) were cannabis-only users. Three drug use groups were created: (a) cannabis-only users who reported using cannabis less than every day during the past 30 days (*n*=253), (b) cannabis-only users who reported using cannabis every day during the past 30 days (*n*=141), and (c) other drug users (*n*=292) who reported using other illegal or nonprescribed drugs either alone or in combination with cannabis. The ASI-Lite also collects information about the number of days in the past 30 days that one used alcohol, although alcohol did not factor into creation of the three drug use groups. The ASI-Lite yields mathematically derived composite severity scores of problem areas in the participant's life commonly affected by substance use, including a drug use severity score, a medical problem score, a psychiatric problem score, and an alcohol problem score.^15,16^ ASI-Lite composite scores range from 0 to 1, with higher scores indicating greater severity of the problem.

Participants also completed the 10-item drug abuse screening test (DAST).^17,18^ Problematic drug use (yes/no) was computed based on a DAST score greater than or equal to 3, a cut-point that has demonstrated accuracy in classifying patients according to *Diagnostic and Statistical Manual of Mental Disorders* (DSM) classification. Self-efficacy for avoiding drug use was measured using a four-item scale assessing confidence in avoiding drug use in various situations, with scores ranging from 1 (low confidence) to 5 (high confidence).^19^

### Analyses

After ruling out problems related to multicollinearity among the correlates, ANOVA and chi-square bivariate analyses were conducted to assess differences among the three drug use groups' sociodemographic characteristics, drug use severity measures, and potential problems related to drug use.

## Results

### Participant characteristics

Overall participant sociodemographic characteristics were reported in a previous article.^14^ Three-fourths of the 686 participants were male. The average age was 36.9 years (SD=13.2). The sample was ethnically diverse, with a third being Latino. Sixty-five percent reported an annual income of less than $10,000 per year. Cannabis was by far the most common drug used (84%). Fifty-seven percent of participants were cannabis-only users. The most common noncannabis drugs of use among other drug users were amphetamines (46.7%), cocaine (21.5%), heroin (18.8%), and opiates (17.9%).

### Differences among drug use groups

Table 1 presents comparisons among nondaily cannabis-only users, daily cannabis-only users, and other drug users. The three drug use groups did not differ with regard to gender, race/ethnicity, marital status, education, employment status, and medical ASI scores. Both groups of cannabis-only users were significantly younger than other drug users, and nondaily cannabis-only users had significantly higher income levels than other drug users. Forty-five percent of the sample overall was identified as having problematic drug use according to the DAST, a percentage that varied greatly by drug use group (∼19% for nondaily cannabis-only, 29% for daily cannabis-only, and 76% for other drug users). Drug ASI severity scores varied significantly by group, with nondaily cannabis-only users having relatively low drug ASI scores, other drug users having relatively high drug ASI scores, and daily cannabis-only users being intermediate. Both groups of cannabis-only users had lower psychiatric and alcohol ASI scores than other drug users, yet did not differ significantly from one another. All groups differed with regard to self-efficacy for drug avoidance. Daily cannabis-only users had the lowest self-efficacy, nondaily cannabis-only users had the highest self-efficacy, and other drug users were intermediate.

**Table 1.** **Comparison of Nondaily Cannabis-Only, Daily Cannabis-Only, and Other Drug Users Visiting Two Large Emergency Departments**

Characteristic	Nondaily cannabis only (*n*=141)	Daily cannabis only (*n*=253)	Other drug users (*n*=292)	*p*
Age, years mean (SD)	36.4 (13.3)	34.9 (12.2)	38.5 (13.3)	0.014^a^
Male, %	76.1	77.8	73.3	ns
Race/ethnicity, %				
Hispanic/Latino	31.3	29.8	36.0	
White non-Hispanic	38.9	30.5	38.8	
Black	25.8	32.6	20.4	
Other	4.0	7.1	4.8	ns
Marital status, %				
Single	85.2	82.3	82.1	
Married	14.8	17.7	17.9	ns
Education, years, mean (SD)	12.2 (2.1)	12.0 (2.4)	12.1 (2.2)	ns
Income, category, mean (SD)	2.0 (1.5)	1.9 (1.4)	1.6 (1.2)	0.014^b^
Employed past 30 days, %	34.9	36.9	29.8	ns
Problematic drug use based on DAST, %	18.8	28.8	75.7	0.000^c^
Drug ASI scores, mean (SD)	0.03 (0.03)	0.09 (0.04)	0.11 (0.10)	0.000^c^
Medical ASI scores, mean (SD)	0.66 (0.20)	0.66 (0.19)	0.66 (0.20)	ns
Psychiatric ASI scores, mean (SD)	0.26 (0.23)	0.26 (0.22)	0.36 (0.25)	0.000^a^
Alcohol ASI scores, mean (SD)	0.09 (0.14)	0.10 (0.14)	0.14 (0.19)	0.001^a^
Self efficacy, mean (SD)	3.8 (1.0)	2.6 (1.2)	3.1 (1.1)	0.000^c^

^a^ Nondaily and daily cannabis-only users significantly different from other drug users.

^b^ Nondaily cannabis-only users significantly different from other drug users.

^c^ All groups significantly different.

ASI, Addiction Severity Index; DAST, drug abuse screening test; ns, not significant at the 0.05 level; SD, standard deviation.

## Discussion

Results suggest that ED patients who use drugs other than cannabis are at particular risk for high drug use severity and concomitant problems such as psychiatric problems and alcohol use severity. However, 19–29% of cannabis-only users were identified as having problematic drug use. Furthermore, daily cannabis-only users fared less well than nondaily cannabis users with regard to drug use severity indicators and self-efficacy for avoiding drug use, even though they were younger as a group.

Although cannabis may be less harmful to individual health relative to other forms of drug use, heavier cannabis use is a concern because it may be associated with mental and physical health problems.^7–10,20^ Cannabis dependence does occur, and individuals often perceive themselves as unable to stop.^21^ Daily cannabis-only users in our study were found to have particularly low confidence in avoiding drug use (compared with nondaily cannabis users and even other drug users). However, there is a distinction between self-efficacy and motivation, perceived importance, and desire to avoid drug use—daily cannabis users may not be motivated or see the importance of avoiding use. Connor et al.^22^ found low cannabis refusal self-efficacy was related to problematic use. Enhancement of motivation and self-efficacy beliefs could be an important target of prevention and treatment. As with other drug use, behavioral interventions and motivational enhancement appear to be useful with problematic cannabis use.^21^ Screening, brief intervention, and referral to treatment (SBIRT) is a promising public health strategy that has been implemented in EDs to reduce risks associated with substance use.^23–25^ Although SBIRT has been shown to be effective for alcohol misuse, its impact on drug use, including cannabis, is less conclusive. However, SBIRT's emphasis on motivation and harm reduction could fit well with cannabis use reduction goals to the degree that many cannabis users express interest in reducing use rather than abstinence.^21^

Possible limitations of the study include not having information about the reason for the ED visit and uncertainty of the degree to which respondents are representative of the overall ED population. Use of cannabis in terms of medical or recreational use was not analyzed, an important distinction since there is some evidence that medical and recreational cannabis users differ in their clinical characteristics.^26^ The quantity used and the frequency of use *per day* were not collected. In addition, reliance on self-reports may have resulted in underestimates of cannabis use and other drug use.

Across the drug use groups, 45% of the ED participants screened positive for problematic drug use, a percentage that varied considerably by type of drug (cannabis vs. other) and frequency of use (daily vs. nondaily). This information may assist emergency medicine providers and social work professionals in matching patients to appropriate intervention. For example, users of drugs other than cannabis (and perhaps heavy, chronic cannabis users) may need referral to specialty services for further assessment. Recently, Konstantopoulos et al.^5^ suggested a clinical decision rule for performing a detailed assessment of ED patients for possible problematic drug use that is based on the type of drug. Because the ED represents an important “opportunistic” venue for harm reduction and intervention efforts with drug users, hospital staff and health educators screening for substance use should be attentive to differences in use patterns among patients, as some adverse consequences may depend on the type and frequency of substances being used.

## Abbreviations Used

ANOVA: analysis of variance
DAST: drug abuse screening test
ED: emergency department
IRB: Institutional Review Board
SBIRT: screening, brief intervention, and referral to treatment
SD: standard deviation
